# Proteomic Signatures of Hippocampal Nonsynaptic and Synaptosome-Enriched Mitochondria in Rats Resilient to Chronic Social Isolation

**DOI:** 10.3390/biom15101358

**Published:** 2025-09-24

**Authors:** Dragana Filipović, Christoph W. Turck

**Affiliations:** 1 Department of Molecular Biology and Endocrinology, VINČA Institute of Nuclear Sciences, National Institute of the Republic of Serbia, University of Belgrade, 11000 Belgrade, Serbia; 2 Key Laboratory of Animal Models and Human Disease Mechanisms of Yunnan Province, KIZ/CUHK Joint Laboratory of Bioresources and Molecular Research in Common Diseases, Kunming Institute of Zoology, Chinese Academy of Sciences Kunming, Kunming 650223, China; turck@psych.mpg.de

**Keywords:** resilience, chronic social isolation, hippocampus, nonsynaptic mitochondria, synaptosome-enriched mitochondria, proteomics

## Abstract

Chronic social isolation (CSIS), a known risk factor for the development of major depressive disorders, is associated with hippocampal dysfunction. In rodent models, CSIS produces two phenotypes: CSIS-susceptible, which develop depressive- and anxiety-like behaviors, and CSIS-resilient, which maintain normal behavior despite stress. However, the biological mechanisms underlying resilience to stress remain elusive. Mitochondria, as central regulators of neuronal energy metabolism and redox balance, are potential mediators of stress susceptibility and resilience. This review summarizes comparative proteomic analyses of hippocampal nonsynaptic mitochondria (NSM) and synaptosome-enriched mitochondria from CSIS-susceptible and CSIS-resilient rats along with controls. In NSM of resilient rats relative to susceptible rats, remodeling enhanced energy production, limited reactive oxygen species, stabilized phosphate transport, and promoted removal of damaged components. Compared with controls, these changes optimized energy production, and selectively downregulated oxidative stress-promoting proteins. Conversely, synaptosome-enriched mitochondria from resilient rats showed downregulation of proteins related to synaptic energy metabolism and redox balance relative to CSIS-susceptible rats, but demonstrated upregulation of bioenergetic and antioxidant enzymes, molecular chaperones, and neuroprotective factors compared with controls. These proteomic signatures both highlight mitochondrial adaptability in promoting stress resilience and identify mitochondria as promising targets for the development of novel antidepressant therapies.

## 1. Introduction

Psychosocial stress is a well-established risk factor for major depressive disorder (MDD), exerting profound effects on both the structure and function of stress-sensitive brain regions such as the hippocampus [1,2,3]. Among the various forms of chronic psychosocial stressors, chronic social isolation (CSIS) represents a particular trigger. Defined as voluntary or involuntary (perceived) disconnection from the social environment, CSIS results in reduced or absent social interactions, and has been consistently linked to the onset and persistence of depressive symptoms in both humans and experimental animals [4,5,6].

Adult male rats exposed to CSIS for six weeks develop behavioral and neuroendocrine changes that closely mirror those seen in human MDD [5,7,8,9]. CSIS has been shown to evoke depressive-like behaviors in rats, evidenced by reduced preference for sucrose, indicative of an impaired sensitivity to reward and anhedonia, one of the core symptoms of depression [10,11,12]. Socially isolated rats also demonstrate behavioral despair, reflected by increased immobility in the forced swim test (FST), which has been linked to depressed mood and/or behavioral helplessness [13]. Anxiety-like behaviors are also prominent, as demonstrated by increased marble-burying activity [14,15], reduced locomotor activity, and increased anxiogenic responses in the elevated plus maze [16], and less time spent in the light compartment of the light–dark box [17]. Notably, animals exhibit individual differences in their behavioral responses to CSIS, with some showing resilience to its depressive and anxiogenic effects [18,19]. Importantly, resilience is not simply the absence of vulnerability but represents a set of active and adaptive biological and psychological processes that counteract the adverse effects of stress [20,21,22,23].

The hippocampus, a highly plastic and stress-sensitive brain region, undergoes profound structural and functional changes in response to environmental and psychological stressors [24,25,26]. Dysfunction of hippocampal neurons is an important contributor to the pathophysiology of MDD, particularly in relation to cognitive impairment and increased stress reactivity [27,28]. Moreover, hippocampal neurotransmitter systems play a role in determining individual sensitivity or resilience to social stress [29]. Conversely, adaptive hippocampal responses, including increased neurogenesis [30,31], improved mitochondrial function and energy metabolism [32], and strengthened synaptic connectivity [33], promote resilience by enabling the maintenance of behavioral and emotional stability under chronic stress. Due to its sensitivity and adaptability, the hippocampus represents a critical region for investigating the cellular mechanisms that underlie individual differences in susceptibility and resilience to stress [34,35].

Mitochondria, central regulators of the cellular stress response, are essential not only for ATP production and energy metabolism, but also for the regulation of complex behaviors, including social interaction [36]. Beyond energy production, mitochondria participate in neurotransmitter metabolism and dynamically respond to extracellular stress signals. Accumulating evidence indicates that mitochondrial dysfunction, reflected by changes in both function and morphology, is a critical factor in the onset and progression of depression, as observed in the brains of MDD patients [37,38,39] and other stress-related psychopathologies [34,40]. According to the mitochondrial dysfunction hypothesis of depression [41], impaired mitochondrial energy production characterized by reduced efficiency of the electron transport chain (ETC), decreased ATP synthesis, and elevated oxidative stress may contribute to the development of depressive symptoms [37,41,42,43].

This dysfunction is particularly complex in neurons, where mitochondria exhibit remarkable compartment-specific specialization. Neuronal mitochondria are not only structurally distinct but also functionally specialized across different subcellular compartments. Additional heterogeneity exists within individual subpopulations based on local conditions and energy demands, enabling them to contribute diverse and adaptive roles in neuronal physiology [44,45,46,47]. Nonsynaptic mitochondria (NSM), predominantly located in soma and dendritic compartments, are closely associated with postsynaptic activity and primarily support anabolic processes. These include protein synthesis and cellular maintenance, which they facilitate through efficient energy generation. NSM display specialized bioenergetic profiles characterized by increased oxidative phosphorylation efficiency, a feature that enables them to meet the continuous energy requirements of several critical processes: dendritic protein trafficking, local mRNA translation, and postsynaptic receptor cycling, all of which are essential for proper neuronal function and synaptic plasticity [48,49]. The localization of NSM near endoplasmic reticulum contact sites further enhances their functional capacity by promoting calcium buffering and lipid synthesis through rapid transfer of ions and lipids. This spatial arrangement supports membrane biogenesis and maintains cellular homeostasis, thereby ensuring effective signaling and metabolic function [50].

In contrast, synaptosomes, isolated nerve terminals comprising intact pre- and postsynaptic membranes, contain synaptic mitochondria, which are primarily of presynaptic origin. These mitochondria provide the ATP required for synaptic vesicle cycling, neurotransmitter release, and other energy-intensive synaptic processes [51,52,53]. Synaptic mitochondria function under distinct bioenergetic demands, necessitating rapid ATP turnover to sustain continuous synaptic vesicle recycling and neurotransmission [54]. Their metabolic flexibility enables quick shifts between glucose and lactate utilization, depending on substrate availability and neuronal activity levels [55].

Structurally, synaptic mitochondria exhibit smaller size and higher surface-to-volume ratio compared to NSM, which enhances their ability to rapidly uptake and release calcium ions. This structural adaptation is crucial for maintaining presynaptic calcium homeostasis and supporting efficient neurotransmitter release during synaptic activity [56,57]. However, this functional specificity underlies their differential sensitivity to stress and disease [58]. Due to their high metabolic demands and exposure to fluctuating calcium levels, synaptic mitochondria are particularly susceptible to dysfunction, which is closely linked to impaired synaptic plasticity and integrity, both hallmarks of depression [59,60].

The implications for neuronal energy balance are profound when synaptic mitochondrial functions become compromised. Under these conditions, neurons must redirect energy from NSM to compensate for the energetic deficit at synapses, potentially compromising essential dendritic maintenance processes and local protein synthesis machinery [61,62]. This metabolic reallocation fundamentally alters cellular energy homeostasis, forcing a detrimental trade-off between immediate synaptic demands and long-term structural integrity [63]. The resulting shift in energy allocation may drive the dendritic atrophy and decreased synaptic connectivity observed in depression, thereby creating a cycle in which impaired energy metabolism further exacerbates synaptic dysfunction [64,65].

Overall, these findings emphasize that mitochondrial subpopulations within neurons exhibit both structural diversity and functional specialization, each serving essential yet distinct physiological roles. Their differential sensitivities to stress highlight the importance of examining mitochondria at the subcellular level, to fully understand their roles in health and disease. Accordingly, this review summarizes comparative proteomic analyses of hippocampal NSM and synaptosome-enriched mitochondria from CSIS-resilient and CSIS-susceptible adult male rats, alongside controls, using untargeted liquid chromatography–tandem mass spectrometry [66,67]. These comprehensive studies identify distinct proteomic signatures and altered biochemical pathways that distinguish resilience from susceptibility to CSIS, providing insights into the cellular mechanisms that protect against depression- and anxiety-like behaviors. Understanding these resilience-associated mechanisms is essential for advancing our knowledge of stress resilience and may pave the way for more precise and targeted therapeutic strategies.

## 2. Proteomic Sample Preparation and Analysis

Hippocampal NSM and synaptosome-enriched mitochondrial fractions were isolated from adult male Wistar rats across three groups: control, CSIS-susceptible, and CSIS-resilient, then prepared for proteomic analysis [66,67]. Following tissue homogenization, samples underwent differential centrifugation through Percoll gradients (15%, 24%, and 40%) to obtain NSM and synaptosome-enriched fractions [68]. Protein concentrations were determined using the Lowry method [69]. Fraction purity was confirmed by Western blotting using compartment-specific markers: α-tubulin (sc-8035, Santa Cruz) for cytosolic fractions, TATA-binding protein (ab51841, Abcam) for nuclear fractions, cytochrome c oxidase subunit IV (COX IV) (Cell Signalling, No. 4850) for mitochondrial fractions, and synaptophysin (SYP) (H-93) (sc-9116, Santa Cruz) for synaptosomal fractions. β-actin (sc-47778, Santa Cruz) served as the loading control.

For gel-based sample preparation, NSM and synaptosome-enriched mitochondrial fractions were separated by SDS-PAGE using NuPAGE 4–12% Bis-Tris gels, stained with Coomassie blue, and excised by gel lane for in-gel processing. Following reduction and alkylation, proteins were digested overnight with trypsin. The resulting peptides were extracted using an acidic acetonitrile solution (1.5% formic acid, 66% acetonitrile), dried, and re-dissolved in 0.1% trifluoroacetic acid before separation using an RSLC nano HPLC system (Dionex) equipped with C18 columns.

Mass spectrometry (MS) analysis was performed by electrospraying peptides into an LTQ Orbitrap XL mass spectrometer (Thermo Scientific) using a Triversa Automate ion source. The resulting MS/MS spectra were searched against the UniProt/SwissProt database using Proteome Discoverer 1.3, with parameters allowing for common variable modifications and one missed cleavage (precursor mass tolerance ± 10 ppm; fragment mass tolerance ± 0.5 Da). Protein identifications were validated using a decoy database at 1% false discovery rate (FDR), retaining only proteins supported by ≥2 unique peptides. Relative protein quantification across the three groups was performed using label-free quantification in Sieve 2.0 software (mass tolerance ± 10 ppm; retention-time shift ± 1 min). Differentially expressed proteins meeting statistical significance criteria (adjusted *p* < 0.05; peptides *p* < 0.01) and showing meaningful fold changes (> 1.2 or < 0.8) were subsequently analyzed for interaction networks using STRING 11.0 based on UniProtKB accession numbers.

## 3. Proteomic Signatures of Hippocampal Nonsynaptic Mitochondria in CSIS-Resilient vs. CSIS-Susceptible Rats

Under CSIS, hippocampal neurons in adult male rats face a delicate balance between energy production and oxidative stress [66]. Consistently, findings in socially isolated mice demonstrated that stress-induced mitochondrial adaptations closely correlate with behavioral outcomes [70,71]. Proteomic analyses of NSM further show that CSIS-resilient rats exhibit specific molecular adaptations that enhance their ability to cope with stress [66], whereas CSIS-susceptible rats display widespread NSM dysfunction relative to controls [14]. These findings suggest that resilience to stress is associated with targeted NSM remodeling that preserves neuronal function under CSIS.

**Figure 1:**
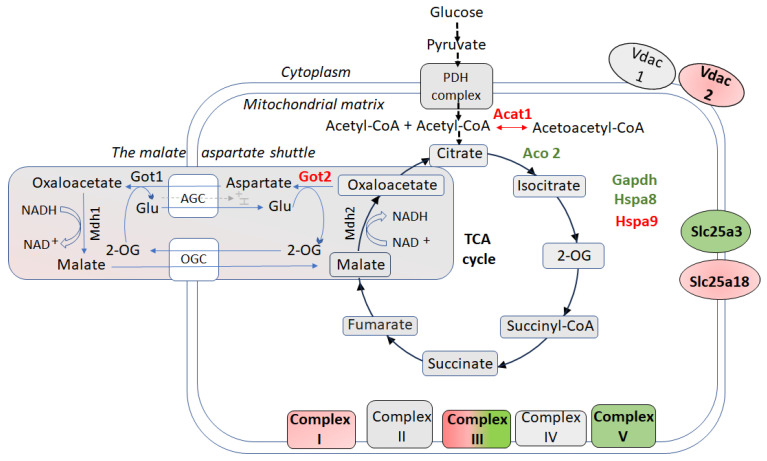
Schematic view of cellular redox metabolism and molecular changes in the hippocampal nonsynaptic mitochondria (NSM) of CSIS-resilient vs. CSIS-susceptible adult male rats. Up or downregulated proteins are highlighted in green and red, respectively. 2-OG–2-oxoglutarate; Acat1–acetyl-CoA acetyltransferase 1; AGC—aspartate/glutamate carrier; Complex I contains Ndufs7 which was downregulated; Complex III showed upregulation of Uqcrc1 and Uqcrc2; Complex V (ATP synthase), showed upregulation of Atp5f1a (α subunit) and Atp5f1b (β subunit); Gapdh—glyceraldehyde-3-phosphate dehydrogenase; Glu—glutamate; Got1—cytosolic aspartate aminotransferase; Got2—mitochondrial aspartate aminotransferase. Mitochondrial aspartate is transported across the inner mitochondrial membrane via the aspartate–glutamate carrier (AGC) in exchange for cytosolic glutamate and a proton (H^+^); Hspa8—heat shock cognate 71 kDa protein; Hspa9—stress-70 protein, mitochondrial; Mdh1—cytosolic malate dehydrogenase; Mdh2—mitochondrial malate dehydrogenase; OGC—malate-2-oxoglutarate carrier; PDH—pyruvate dehydrogenase; Slc25a18—solute carrier family 25 member 18; Vdac1—voltage-dependent anion channel 1; Vdac2—voltage-dependent anion channel 2.

A hallmark of the resilient phenotype was the regulation of key metabolic pathways, including the TCA cycle, ETC, and the oxidative phosphorylation [66] (Figure 1). As detailed in Table S1, multiple proteins within these pathways showed significant differential expression, reflecting enhanced mitochondrial bioenergetic capacity in stress-resilient animals. The TCA cycle enzyme aconitase 2 (Aco2), which catalyzes the conversion of citrate to isocitrate in the TCA cycle [72], was upregulated in CSIS-resilient rats, potentially enhancing mitochondrial ATP production and stabilizing mitochondrial DNA [73]. Alongside Aco2, specific ETC components such as ubiquinol-cytochrome c reductase core protein 2 (Uqcrc2, complex III) and mitochondrial ATP synthase subunits α and β (Atp5f1a/b) were also upregulated, supporting efficient electron transport, maintaining a proton gradient, and strengthening oxidative phosphorylation capacity to support the high energy demands required for stress adaptation [66]. Consistently, recent findings demonstrated upregulation of some subunits of complex I, II, and IV of the ETC in the ventral hippocampus of resilient rats exposed to chronic mild stress [74]. Interestingly, other ETC subunits in CSIS-resilient rats, such as NADH:ubiquinone oxidoreductase (Ndufs7, complex I) and ubiquinol-cytochrome c reductase core protein 1 (Uqcrc1, complex III), were downregulated, likely limiting electron leakage and ROS production, while maintaining energy efficiency, thereby providing a molecular basis for adaptive response to CSIS. Importantly, while these findings are based on protein expression changes, the functional outcomes of mitochondrial metabolism depend not only on protein abundance but also on the enzymatic activity and post-translational modifications of these mitochondrial proteins [75].

A previous proteomic study demonstrated that CSIS-susceptible adult male rats, compared to controls, exhibited downregulation of the majority of hippocampal NSM proteins, particularly those involved in the TCA cycle and oxidative phosphorylation. This pattern reflects mitochondrial dysfunction, reduced ATP production, and impaired stress-coping capacity [14]. Supporting this findings, socially isolated mice displaying anxiety-like behavior and mild cognitive impairment [71,76] showed a 43% reduction in ATP levels, alongside an approximately 52% reduction in succinate dehydrogenase (Complex II) capacity [70].

A pivotal component of this protective network was glyceraldehyde-3-phosphate dehydrogenase (Gapdh), showing a four-fold increase in CSIS-resilient rats. Although primarily known as a cytosolic glycolytic enzyme, Gapdh can translocate to mitochondria under oxidative stress [77,78], where post-translational modifications such as phosphorylation and oxidation critically regulate its mitochondrial functions and broader cellular stress responses [78,79,80]. Within mitochondria, Gapdh contributes to micro-mitophagy, a selective process in which discrete portions of the outer mitochondrial membrane are targeted to lysosome-like compartments without engulfing the entire organelle. This targeted removal of damaged mitochondrial segments helps maintain the integrity and functionality of the mitochondrial network. Importantly, this protective role of Gapdh is independent of its glycolytic enzymatic activity and may protect neurons from caspase-independent cell death [78,79].

In CSIS-resilient rats, the upregulation of heat shock cognate 71 kDa protein (Hspa8), a central molecular chaperone [81], may suggest activation of chaperone-mediated autophagy, a mechanism supporting mitochondrial quality control [82]. Hspa8 selectively recognizes and binds Gapdh [83], targeting it along with potentially associated mitochondrial fragments and directing them toward lysosomal degradation. This targeted removal preserves mitochondrial integrity, which is essential for maintaining neuronal activity, as both autophagy and mitophagy are essential for sustaining mitochondrial homeostasis [84]. In contrast, hippocampal neurons from mice exposed to chronic unpredictable mild stress (CUMS), an established animal model of depression [85], display numerous autophagosomes, but lack mitophagosomes or mitolysosomes, suggesting that CUMS impairs mitophagy [86]. Interestingly, other mitochondrial chaperones such as Hspa9 were downregulated, suggesting that mitochondrial chaperone systems undergo selective regulation depending on their specific roles in cellular stress signaling and quality control mechanisms.

In addition, the mitochondrial phosphate carrier, solute carrier family 25 member 3 (Slc25a3), was upregulated ten-fold in resilient rats, potentially enhancing the import of inorganic phosphate into the mitochondrial matrix to support ATP synthase activity [87,88]. Although changes in Slc25a3 abundance have been reported in a mouse model of high anxiety-related behavior [89], and in parvalbumin-positive interneurons from schizophrenia patients [90], the observed upregulation should be interpreted in the context of enhanced mitochondrial energy metabolism rather than direct behavioral modulation.

Conversely, certain mitochondrial transporters and metabolic enzyme, including voltage-dependent anion channel 2 (Vdac2), mitochondrial glutamate carrier 2 (Slc25a18) [91], and glutamate oxaloacetate transaminase 2 (Got2), components of the malate–aspartate shuttle, were downregulated (Figure 1), limiting ions and metabolites exchange, mitochondrial glutamate uptake, and NADH-driven oxidative phosphorylation. These adjustments likely mitigate excitotoxicity and minimize oxidative stress. Further, acetyl-CoA acetyltransferase 1 (Acat1), involved in ketone body metabolism (reversible conversion between acetoacetyl-CoA and two acetyl-CoA molecules) and the fatty acid β-oxidation cycle [92] was also downregulated, favoring glucose-driven oxidative phosphorylation and further stabilizing energy homeostasis. Together, these coordinated changes in mitochondrial metabolism and transport support neuronal integrity and function under CSIS, emphasizing the pivotal role of NSM adaptability in supporting neurobiological resilience.

## 4. Proteomic Signatures of Hippocampal Nonsynaptic Mitochondria in CSIS-Resilient vs. Control Rats

Resilient rats resisted the effects of CSIS, maintaining sucrose intake and immobility in the FST at levels comparable to controls [66,67]. In control rats, hippocampal NSM exhibited steady-state protein levels, maintaining baseline amounts of TCA cycle enzymes, ETC components, and protein quality-control machinery, to support routine energy demands. Although behaviorally indistinguishable from controls, CSIS-resilient rats exhibited a remodeling of the NSM proteome, indicative of a stress-adapted phenotype, with the involved proteins detailed in Table S2.

**Figure 2:**
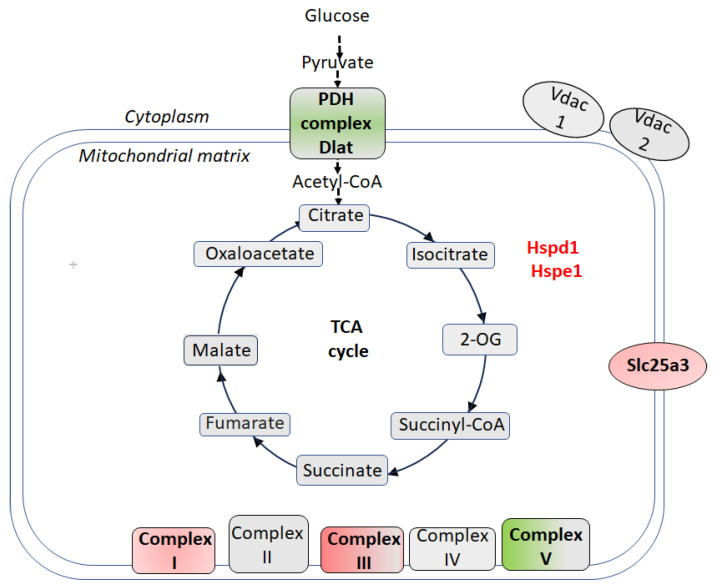
Schematic view of cellular redox metabolism and molecular changes in the hippocampal nonsynaptic mitochondria (NSM) of CSIS-resilient vs. control adult male rats. Up or downregulated proteins are highlighted in green and red, respectively. Atp5f1a—α subunit of mitochondrial ATP synthase (Complex V); Dlat—dihydrolipoyllysine-residue acetyltransferase component of pyruvate dehydrogenase (PDH) complex, mitochondrial; Hspd1 (60 kDa) and Hspe1 (10 kDa)—mitochondrial chaperones; Ndufs7—NADH:ubiquinone oxidoreductase core subunit S7, which is a core component of Complex I; Slc25a3—solute carrier family 25 member 3; Uqcrc2—ubiquinol-cytochrome c reductase core protein 2, a component of Complex III.

Central to this adaptation was the upregulation of dihydrolipoyllysine-residue acetyltransferase component of pyruvate dehydrogenase (PDH) complex, mitochondrial (Dlat)**,** and Atp5f1a, a catalytic subunit of Complex V (ATP synthase) [66] (Figure 2). Dlat catalyzes the formation of acetyl-CoA from acetyl-lipoamide and CoA, linking glycolysis to the TCA cycle [93]. Elevated Dlat levels indicate an enhanced capacity to channel glycolytic intermediates into the TCA cycle, enhancing substrate availability for energy production. Concurrent upregulation of Atp5f1a suggests that increased carbon flux from the TCA cycle (e.g., via Dlat) is efficiently converted and coupled to ATP generation through oxidative phosphorylation. These changes likely improve metabolic efficiency and energy supply, enabling neurons in CSIS-resilient rats to sustain function under CSIS-induced energetic stress.

Interestingly, this upregulation is accompanied by the selective downregulation of other mitochondrial proteins. Levels of mitochondrial phosphate carrier Slc25a3, Ndufs7 (a core subunit of Complex I), Uqcrc2 (Complex III), and chaperones Hspd1 (60 kDa) and Hspe1 (10 kDa) involved in protein folding and quality control [93,94] were downregulated in CSIS-resilient rats relative to controls. This pattern reflects a dual adaptive strategy: lower chaperone abundance may indicate reduced mitochondrial stress and repair demand, while downregulation of Complexes I and III subunits, as a major sources of ROS during electron transport [95,96], likely minimize oxidative damage. Additionally, reduced levels of Slc25a3 may help fine-tune phosphate import ensuring efficient ATP synthesis without overloading the system.

The functional significance of this adaptation is highlighted by fold changes comparisons across phenotypes. Uqcrc2 shows a 0.16-fold change in CSIS-resilient vs. control rats but a 2.89-fold change in CSIS-resilient vs. CSIS-susceptible rats. Likewise, Slc25a3 shifts from 0.20-fold (resilient vs. control) to 10.54-fold (resilient vs. susceptible). These contrasting patterns reveal that NSM remodeling in CSIS-resilient rats is not a passive baseline feature but an active, regulated response to CSIS, minimizing ROS production while directing resources toward efficient energy generation.

## 5. Proteomic Signatures of Hippocampal Synaptosome-Enriched Mitochondria in CSIS-Resilient vs. CSIS-Susceptible Rats

Synaptic transmission is an energy-demanding process, that depends critically on continuous ATP production to support neurotransmitter release, synaptic vesicle recycling, and ion gradient maintenance. Mitochondria play a central role in this process by producing over 90% of all neuronal ATP, required for various steps in the synaptic vesicle cycle, including vesicle filling, mobilization, and recycling [97]. Synaptosomes, which are isolated synaptic terminals [98], contain synaptic mitochondria that are strategically positioned near synaptic vesicles, ensuring efficient ATP delivery to sustain rapid neurotransmitter release during periods of high neuronal activity [99,100]. Furthermore, these mitochondria also regulate calcium homeostasis and signaling, both essential for proper synaptic function [101]. Proteomic analyses of synaptosomal mitochondria have revealed alterations in energy metabolism, mitochondrial transport, oxidative stress, and neurotransmission in anxiety-related behavior [89], highlighting the value of these profiles in understanding stress adaptation and resilience.

**Figure 3:**
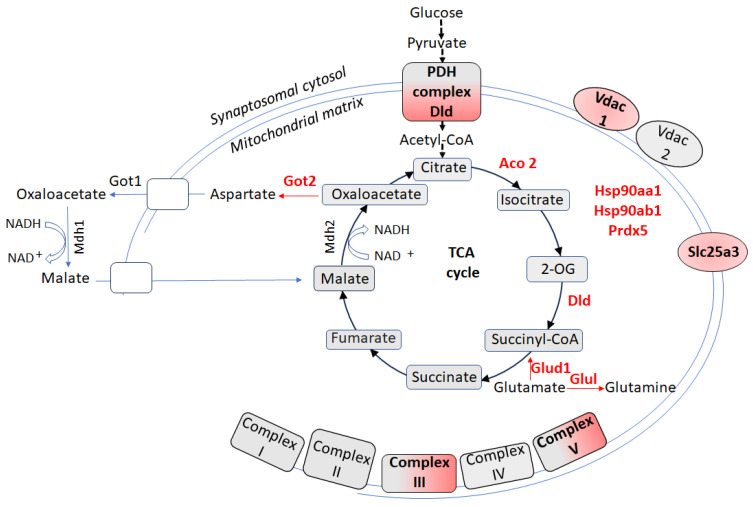
Schematic view of cellular redox metabolism and molecular changes in the hippocampal synaptosome-enriched mitochondria of CSIS-resilient vs. CSIS-susceptible adult male rats. Up or downregulated proteins are highlighted in green and red, respectively. Atp5f1b—β subunit of mitochondrial ATP synthase (Complex V); Uqcrc2 (Complex III); Dld—dihydrolipoamide dehydrogenase as the E3 subunit of the pyruvate dehydrogenase complex (PDH); Glul—glutamine synthetase; Glud1—glutamate dehydrogenase; Got2—glutamate oxaloacetate transaminase 2; Hsp90—heat shock proteins alpha and beta (encoded by the gene Hsp90aa1 and Hsp90ab1); Prdx5—peroxiredoxin-5; Slc25a3—solute carrier family 25 member 3; Vdac1—voltage-dependent anion-selective channel protein 1.

Comparative proteomic analyses of synaptosome-enriched mitochondria in CSIS-resilient rats reveal predominantly downregulated proteins related to energy metabolism compared to CSIS-susceptible rats [67] (Figure 3), with all involved proteins detailed in Table S3. Specifically, Aco2 and Dld (dihydrolipoamide dehydrogenase) protein levels were reduced in the resilient group. Dld, a flavoprotein, serving as the E3 component of both pyruvate dehydrogenase (PDH) and 2-oxoglutarate dehydrogenase complexes, links glycolysis to the TCA cycle by converting pyruvate to acetyl-CoA and promoting TCA cycle progression via 2-oxoglutarate to succinyl-CoA conversion [102].

Components of oxidative phosphorylation, including Uqcrc2 (complex III) and Atp5f1b (ATP synthase, β subunit), were also downregulated, suggesting a suppression of mitochondrial respiration and ATP synthesis within the synaptic compartment. This contrasts with NSM, where energy production was upregulated in CSIS-resilient rats, reflecting a compartment-specific metabolic adaptation. The differential regulation between NSM and synaptosome-enriched mitochondria likely reflects their distinct functional roles: synaptosome-enriched mitochondria primarily support rapid local energy demands and calcium regulation, while NSM support broader cellular energetics. This selective downregulation in synaptosomes may limit ROS production, thereby protecting synapses from excitotoxic stress during CSIS.

Additional adaptive changes included downregulation of Vdac1, decreasing metabolites and ion transport across the outer mitochondrial membrane, and Slc25a3, which couples ADP phosphorylation to ATP synthesis, further indicating lowered synaptic energy production [67]. Proteases regulators Hsp90α and Hsp90β [103,104,105] were also reduced, suggesting either less proteotoxic stress or energy-efficient protective strategies, involving enhanced autophagy or mitochondrial quality control [106,107]. The antioxidant enzyme peroxiredoxin 5 (Prdx5), critical for peroxide detoxification, was similarly downregulated, supporting the notion of reduced oxidative stress in resilient rats [108].

Similarly, enzymes linking glutamate metabolism to mitochondrial function, including glutamate oxaloacetate transaminase 2 (Got2), a component of the malate–aspartate shuttle, glutamine synthetase (Glul) that catalyzes the ATP-dependent conversion of glutamate and ammonia to glutamine, and glutamate dehydrogenase 1 (Glud1), which catalyzes the conversion of glutamate to 2-oxoglutarate, were downregulated in CSIS-resilient rats. This suggests a decreased reliance on glutamate-driven mitochondrial metabolism within synaptosome-enriched mitochondria. Such compartment-specific adaptations likely limit ROS production, preserve synaptic integrity, and contribute to stress resilience, contrasting with the enhanced energy production observed in NSM. Moreover, elucidating synaptic regulation provides insight into the mechanisms of stress resilience, reveals protective pathways against depression, and identifies novel targets for pharmacological intervention.

## 6. Proteomic Signatures of Hippocampal Synaptosome-Enriched Mitochondria in CSIS-Resilient vs. Control Rats

**Figure 4:**
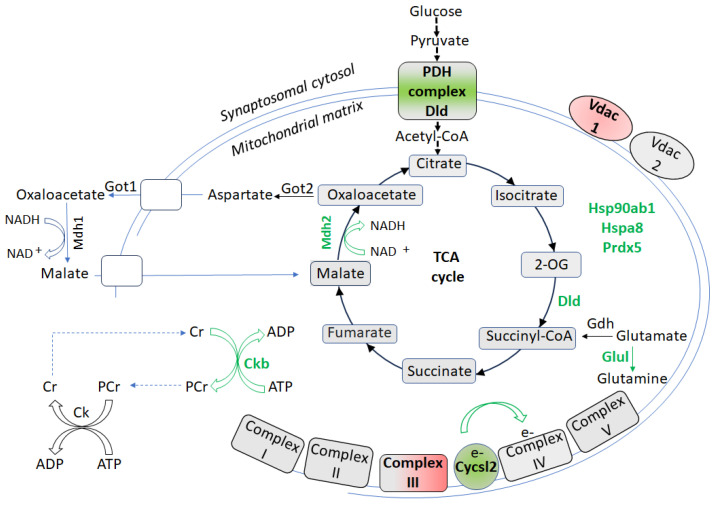
Schematic view of cellular redox metabolism and molecular changes in the hippocampal synaptosome-enriched mitochondria of CSIS-resilient vs. control adult male rats. Up or downregulated proteins are highlighted in green and red, respectively. Ckb—creatine kinase B-type; Uqcrc2 (Complex III); Dld—dihydrolipoamide dehydrogenase; Glul—glutamine synthetase; Hsp90ab1—heat shock protein 90-beta; Hspa8—heat shock cognate 71 kDa protein; Mdh1—malate dehydrogenase 1, cytoplasmic; Mdh2—malate dehydrogenase 2, mitochondrial; Prdx5—peroxiredoxin-5; Vdac1—voltage-dependent anion-selective channel protein 1; Vdac2—voltage-dependent anion-selective channel protein 2.

Although CSIS-resilient rats showed no behavioral differences compared to controls, their synaptosome-enriched mitochondria exhibited distinct proteomic adaptations (Figure 4), characterized by upregulation of proteins that support proteostasis, energy metabolism, redox balance, and neuroprotection [67]. Table S4 presents the list of proteins involved in these adaptive changes. Molecular chaperones, such as Hsp90ab1 and Hspa8, likely facilitate proper protein folding, mitochondrial trafficking to active synapses, and local mitophagy to prevent accumulation of dysfunctional mitochondria [82,109,110,111].

Enzymes involved in mitochondrial bioenergetics, such as mitochondrial malate dehydrogenase (Mdh2) and Dld were upregulated, likely enhancing TCA cycle activity, NADH generation, and malate–aspartate shuttle function, thereby sustaining oxidative phosphorylation and redox homeostasis. Upregulation of creatine kinase B-type (Ckb) may further improve ATP buffering through phosphocreatine metabolism and modulate AKT signaling to limit mitochondrial calcium uptake, preventing permeability transition pore opening and preserving overall mitochondrial integrity [112]. Moreover, mitochondrial Ck activity contributes to antioxidant protection by facilitating ADP recycling [113]. Cytochrome c oxidase subunit-like 2 (CycsL2) upregulation likely improves electron transfer efficiency within Complex IV, further promoting mitochondrial respiration. These bioenergetic adaptations likely serve as neuroprotective responses to CSIS by boosting ATP synthesis and maintaining energy homeostasis [114].

Antioxidant and neuroprotective systems were also enhanced. Prdx5 upregulation strengthens mitochondrial antioxidant defenses by reducing hydrogen peroxide and alkyl hydroperoxides, mitigating ROS-induced damage [115]. The neuroprotective enzyme Glul was also upregulated, supporting glutamate detoxification, preventing excitotoxicity and maintaining neurotransmitter balance [116,117]. Glutamine itself has demonstrated antidepressant effects in a chronic immobilization stress-induced mouse model of depression [118]. Targeting Glul activation thus represents a promising therapeutic strategy for MDD [119]. Conversely, reduced Glul protein levels or activity may lead to elevated glutamate levels, impaired clearance, and heightened risk of excitotoxicity [120].

Interestingly, only Uqcrc2 and Vdac1 were uniquely downregulated in the CSIS-resilient group compared to both controls and CSIS-susceptible rats, suggesting targeted suppression of these proteins. This may prevent the overproduction of ROS or excitotoxic stress observed in CSIS-susceptible rats. Overall, these proteomic changes reflect a resilience-associated mitochondrial proteomic adaptation that enhances energy metabolism, antioxidant capacity, and synaptic stability in CSIS-resilient rats compared to controls.

## 7. Conclusions and Future Directions

Resilience to CSIS is underpinned by compartment-specific remodeling of the mitochondrial proteome. In NSM, resilient rats show selective upregulation of proteins related to TCA cycle enzymes, ETC components, and quality-control proteins, likely enhancing basal bioenergetic capacity and metabolic flexibility while promoting the clearance of damaged mitochondrial components. This represents an adaptive response distinct from controls that maximizes energy efficiency while limiting oxidative stress. Synaptosome-enriched mitochondria from resilient rats exhibit a different strategy through selective downregulation of oxidative phosphorylation and glutamate metabolism, thereby reducing ROS generation and excitotoxic stress. Concurrently, compared to controls, resilient synaptosome-enriched mitochondria upregulate molecular chaperones, bioenergetic enzymes, creatine kinase, and antioxidant systems, supporting synaptic proteostasis, reinforcing redox defenses, and safeguarding mitochondrial function. By balancing reliable energy supply and protection against metabolic overload at the synapse, this mitochondrial proteome remodeling provides the cellular foundation for stress coping.

The differentially expressed proteins identified in both mitochondrial compartments of resilient rats may represent potential novel therapeutic targets for MDD. Among these, Gapdh, Slc25a3, and Prdx5 are of particular interest. Gapdh’s dual role in glycolysis and oxidative stress response supports emerging metabolic frameworks for understanding depression, though clinical translation remains unproven. Slc25a3 (mitochondrial phosphate carrier), and Prdx5 represent components of mitochondrial bioenergetics and antioxidant defense systems that may contribute to stress resilience, pending further validation in clinical populations.

Nevertheless, translating these hippocampal proteomic findings to clinical application remains challenging. Species-specific regulation of protein expression and the pronounced heterogeneity of MDD demand rigorous validation across diverse human populations. While current therapeutic paradigms, including monoaminergic modulation, neurotrophic enhancement, and anti-inflammatory approaches, provide a conceptual framework for evaluating these candidates, confirming their biomarker relevance and druggability will require longitudinal human studies, high-resolution imaging, and functional assays.

Future studies should also clarify which mitochondrial changes associated with resilience to CSIS reflect adaptive compensatory responses to dysfunctions in other cellular systems, and which changes indicate direct mitochondrial impairment due to stress. A comprehensive assessment of mitochondrial biogenesis, mitophagy, fusion and fission dynamics, subcellular distribution, and morphological characteristics will be essential [121]. Given that chronic stress induces region-specific alterations in mitochondrial function [122], detailed mitochondrial analyses across multiple brain regions implicated in resilience to stress are warranted [123]. Moreover, integrating multi-omics datasets (e.g., metabolomics and transcriptomics) and employing patient-derived neuronal models or organoids will also be essential for bridging the gap between preclinical proteomics discoveries and precision therapeutics for MDD.

## Data Availability

Data sharing is not applicable to this article as no datasets were generated or analyzed during the current study.

## Supplementary Materials

The following supporting information can be downloaded at: https://www.mdpi.com/article/10.3390/biom15101358/s1, Table S1: Differentially expressed proteins in hippocampal nonsynaptic mitochondria (NSM) of CSIS-resilient vs. CSIS-susceptible rats; Table S2: Differentially expressed proteins in hippocampal nonsynaptic mitochondria (NSM) of CSIS-resilient vs. control rats; Table S3: Differentially expressed proteins in hippocampal synaptosome-enriched mitochondria of CSIS-resilient vs. CSIS-susceptible rats; Differentially expressed proteins in hippocampal synaptosome-enriched mitochondria of CSIS-resilient vs. control rats; Table S4: Differentially expressed proteins in hippocampal synaptosome-enriched mitochondria of CSIS-resilient vs. control rats.
